# Patent ductus arteriosus shunting direction and diameter predict inpatient outcomes in newborns with congenital diaphragmatic hernia

**DOI:** 10.3389/fped.2023.1272052

**Published:** 2023-10-31

**Authors:** Min Bao, Tao Wu, Jinghui Guo, Ying Wang, Aimei Cao, Chao Liu, Yandong Wei, Chunhua Zheng, Lin Shi, Lishuang Ma

**Affiliations:** ^1^ Department of Cardiology, Children's Hospital in Capital Institute of Pediatrics, Beijing, China; ^2^ Department of Pediatric and Neonatal Surgery, Children's Hospital in Capital Institute of Pediatrics, Beijing, China

**Keywords:** congenital diaphragmatic hernia, echocardiography, neonatal outcomes, patent ductous arteriosus, pulmonary hypertension

## Abstract

**Objective:**

To evaluate whether the patent ductus arteriosus (PDA) can serve as a predictive factor for inpatient outcomes in congenital diaphragmatic hernia (CDH) patients.

**Methods:**

A retrospective cohort study was conducted on 59 CDH patients at the Capital Institute of Pediatrics from January 2020 to August 2022. Echocardiography was performed at least three times: within 2–3 h after birth, pre-operatively, and post-operatively of CDH surgery. Based on the direction of the PDA shunt in the first echocardiogram, patients were classified into three groups: left-to-right shunting or closed PDA (L-R), bi-directional shunting, and right-to-left shunting (R-L).

**Results:**

The mortality rate was 15.3% (9/59), with all non-survivors having R-L shunting and group mortality of 39.1% (9/23). The direction of the PDA shunt was significantly associated with the duration of ventilation and length of hospital stay (*p *< 0.05). Decreased PDA diameter or pre-operative shunting direction change towards L-R or bi-directional shunting were associated with higher survival rates, while increased PDA diameter or continuous R-L shunting were associated with higher mortality rates. Pre-operative PDA shunt direction, PDA size after birth and before surgery, gestational age of diagnosis, and shortening fraction before surgery were significantly correlated with patient outcomes. The direction of the preoperative PDA shunt was the most relevant factor among these relationships (*p *= 0.009, OR 20.6, CI 2.2∼196.1).

**Conclusion:**

Our findings highlight the importance of monitoring changes in PDA shunt directionality and diameter in the early stage after birth, as these parameters may serve as valuable predictors of patient outcomes.

## 1. Introduction

Congenital diaphragmatic hernia (CDH) is a rare but serious birth defect that affects 2.5–3.8 cases per 10,000 live and stillbirths (1). CDH is characterized by an anatomical defect in the diaphragm that allows abdominal contents to herniate into the thoracic cavity, leading to pulmonary hypoplasia, abnormal pulmonary vascular development, and vasoreactivity. Pulmonary hypertension (PH) often develops a consequence, and its severity has been found to be directly correlated with morbidity and mortality in CDH patients (1–4). Early diagnosis and management of PH is crucial to improve patient outcomes, and PH severity can serve as a prognostic factor in CDH management (5, 6).

Traditionally, right heart catheterization is considered the gold standard for measuring mean pulmonary arterial pressure (mPAP) to diagnose PH. However, due to its invasive nature, echocardiography is often used as a first-line tool for bedside assessment (7). To classify the degree of PH in infants, mPAP is compared to systemic systolic blood pressure, and the severity is categorized as no/mild, moderate, or severe (8). While tricuspid regurgitation (TR) peak velocity is commonly used to calculate pulmonary arterial pressure (PAp) by echocardiography (7, 9), this method may not always be feasible (10). Other echocardiography methods, such as assessing the patent ductus arteriosus (PDA) (11), ventricular septum configuration (12), and pulmonary artery flow patten (13), have been proposed to measure PAp.

In this study, we aim to investigate whether the direction and diameter of blood flow through the PDA after birth can predict inpatient outcomes in CDH patients. Previous research has not solely used the PDA approach to monitor PAp in CDH patients and analyze the relationship between PDA and inpatient outcomes. We believe that this study can provide valuable insights into the use of the PDA approach for assessing PAp in CDH patients and its potential as a predictor of inpatient outcomes.

## 2. Materials and methods

### 2.1. Study design and population

A retrospective cohort study was conducted at the Capital Institute of Pediatrics from January 2020 to August 2022. The study protocol was approved by the Institutional Ethics Review Board, and patient informed consent was waived in accordance with the study's retrospective design (SHERLLM2022035).

The study included 59 newborns prenatally diagnosed with CDH who were immediately intubated after birth and transferred to our hospital for clinical assessment. High-frequency oscillatory ventilation was used for patients with refractory hypercapnia or high peak inspiratory pressures. Dopamine, dobutamine, epinephrine, and norepinephrine were used to improve poor perfusion and keep the blood pressure in normal range (mostly 50–65 mmHg in systolic pressure) (14). Prostaglandin E1 was not used and milrinone almost never done. Surgical repair for CDH was performed following clinical stabilization. The entire treatment process mainly followed Canadian guidelines (15).

To assess pulmonary hypertension (PH) and guide treatment decisions, at least three echocardiography evaluations were conducted: (1) within 1–2 h after arriving at our hospital (actually most in 2–3 h after birth), (2) pre-operative (mostly the second day, actually after birth within 24 h), and (3) repeated post-operative daily echocardiography if the PDA shunting was right-to-left (R-L) or every 2–3 days if shunting was bi-directional or left-to right (L-R) until the PDA was closed. These patients with CDH were evaluated by doctors with experienced skills due to their critical condition.

If R-L shunting was revealed after the first echocardiography, and medical treatment for PH including inhaled nitric oxide, sildenafil, and Treprostinil was administered.

### 2.2. Patient groups and outcomes

Transthoracic echocardiography was performed by a CX50 ultrasound machine equipped with an S12-4 probe operating at a frequency of 12-4 MHz or an S8-3 Probe operating at a frequency of 8-3 MHz (Intelligent Echocardiography System 33, Philips). Based on the patency and shunting direction of the PDA identified in the first 24-h echocardiography, the CDH newborns were classified into three groups: Left-to-right (L-R) shunting, which was described as a PDA shunting in a L-R shunting or closed PDA; Bi-directional shunting, which was identified by a PDA shunting with both directions; Right-to-left (R-L) shunting, which was described as PDA shunting in a pure R-L shunting or little left to right shunt (5%). These groupings were based on a standardized protocol for PDA shunting classification.

The primary outcome of this study was in-hospital mortality rate of newborns with CDH. Secondary outcomes included age at surgical repair, ventilator days, hospital length of stay, and the changes in PDA size and shunting direction before and after surgical repair. Furthermore, this study aimed to compare and analyze the variation in these outcomes between patients who survived and those who did not, and to examine the potential correlation between the outcomes and PDA.

### 2.3. Statistical analysis

Categorical variables were presented as frequency distributions, while continuous data were expressed as mean with standard deviation or median with interquartile range. Categorical data were compared using the *χ*2 or Fisher's exact test, while continuous data were analyzed using ANOVA or Kruskal-Wallis tests. The correlation between variables was assessed using Spearman's tests and univariate binary logistic regression. All statistical analyses were performed using SPSS Statistics version 22.0. A *p*-value of less than 0.05 was considered statistically significant.

## 3. Results

### 3.1. Patient characteristics

The study included 59 newborns with prenatal CDH diagnosis. The average birthweight of the cohort was 3,015.4g ± 546.2 in 1,000 g to 3,550 g. There were 33 males (55.9%) and 26 females (44.1%). The median gestational age at the time of prenatal diagnosis was 24 weeks (IQR: 22.0–30.0 weeks), and the median gestational age at birth was 37.7 weeks (IQR: 37.1 to 38.1 weeks). The median surgery age is 1.0 day (IQR 1.0–2.0 days). The majority of cases had left-sided CDH (72.9%), while 25.4% had right-sided CDH, and one patient had bilateral CDH. Other cardiac anomalies, including patent foramen ovale (PFO), atrial septal defect (ASD), ventricular septal defect (VSD), and aortic stenosis (AS), were also observed in some cases (Table 1).

**Table 1:** Patient characteristics stratified by the directionality of PDA shunting.

	No PDA or L-R shunting	Bidirectional shunting	R-L shunting	*p*-value
Total Number (*n*, %)	8 (13.5%)	28 (47.5%)	23 (39.0%)	
Male/female ratio	8/0	13/15	11/12	
Mortality (*n*, %)	0 (0.0%)	0 (0.0%)	9 (39.1%)	
Gestational age of diagnosis (w)	28.0 (20.5–36.0)	24.5 (23.3–31.0)	24.0 (22.0–26.0)	0.210
Gestational age of birth, weeks, median (IQR)	37.7 (35.3–38.2)	37.6 (37.1–38.4)	37.7 (37.1–38.0)	0.731
Birth weight (g)	2,951.9 ± 753.5	2,957.0 ± 730.1	2,940.4 ± 594.7	0.775
Diaphragmatic defect side				
Left/Right/Bilateral-side(*n*)	7/1/0	22/5/1	14/9/0	0.311
Age of first Echo (h, IQR)	3.0 (2.0–15.0)	2.0 (1.5–3.0)	2.0 (1.5–3.0)	0.311
VIS after birth (median, IQR)	5.0 (0–6.9)	5.0 (4.0–10.0)	5.0 (5.0–8.2)	0.524
VIS before surgery (median, IQR)	5.0 (5.0–14.5)	10.0 (5.3–13.0)	18.0 (9.5–35)	0.049
VIS after surgery (median, IQR)	7.0 (5.0–15.5)	16.0 (8.0- 29.0)	15.0 (9.5–32.5)^a^	0.124
PH treatment (no. None/NO/NO + Treprostinil)	5/2/1	15/8/5	1/5/17	
PA/AO	1.0 ± 0.2	1.1 ± 0.3	1.2 ± 0.2	0.061
Shortening fraction after birth (%)	36.1 ± 4.3	35.8 ± 3.4	34.4 ± 4.5	0.484
Tricuspid Regurgitation (*n*, %)	3 (37.5%)	11 (39.3%)	17 (73.9%)	0.02
PDA size after birth (median, IQR)	0 (0–1.8)	4.0 (3.3–4.9)	5.0 (4.0–5.2)	0.001
Other cardiac anomalies (*n*.)				
PFO	7	23	19	
ASD	1	5	5	
VSD		1	2	
AS			1	

ASD, Atrial septal defect; AS, Aortic stenosis; AO, aortic artery; L-R, Left-to-right; PDA, Patent ductus arteriosus; PA, Pulmonary artery; PFO, Patent foramen ovale; R-L, Right-to-left; VSD, Ventricular septal defect; VIS, vasoactive-inotropic score. NO, nitric oxide.

^a^ Patients in this group was excluded the non-survived cases.

### 3.2. Patient outcomes

The mortality rate of the entire cohort was 15.3% (9/59), with all deaths occurring in the R-L shunting group, which had a group mortality rate of 39.1% (9/23). There was a significant difference in vasoactive-inotropic score (VIS), and the R-L shunting group before the surgery utilized the highest vasoactive medicine.

Tricuspid regurgitation was detected in 31 out of 59 (52.5%) cases, with the R-L shunting group having the highest detection rate. PDA patency was assessed in 54 out of 59 (91.5%) cases within 24 h after birth, and the L-R shunting group had the smallest median PDA diameter of 0 mm (IQR 0–1.8 mm), while the R-L shunting group had the largest median diameter of 5.0 mm (IQR 4.0–5.2 mm). No significant differences were observed among the PDA shunting groups in terms of gestation age, birth weight, shortening fraction after birth, and PA/AO ratio.

### 3.3. PDA findings at the three timepoints

In the L-R shunting group, the median times of the three echocardiography were 3 h, 28.5 h and 90 h, respectively, with 3, 1 and 0 open PDAs. In the bidirectional shunting group, the median ages of the three examinations were 2 h, 22 h and 46.5 h, and the number of open PDAs was 28, 25 and 16, respectively. The PDAs changed from bidirectional to L-R shunts in 14 cases preoperatively, maintained bidirectional shunts in 14 cases, while postoperatively they were L-R shunts or closed in 22 cases, bidirectional shunts in 4 cases, and R-L shunts in 2 cases. In the R-L shunting group, the median age of the three groups examined was 2 h, 22.3 h and 47 h, the number of open PDAs was 23, 23 and 19, and the size of the PDAs was 5.0 mm, 4.0 mm and 3.3 mm, respectively. The PDAs changed preoperatively to become left-to-right shunts in 3 cases, bi-directional shunts in 8 cases, and remained right-to-left shunts in 12 cases, and postoperatively, two cases of left-to-right shunts were found to be open, 2 cases were closed and 10 cases were bi-directional, 5 cases right to left. There was no difference in LV systolic function (Table 2).

**Table 2:** A table with the PDA findings at the three timepoints.

	First Echo after birth	Echo Before surgery	Echo After surgery	*p*-value
PDA in L-R group (*n* = 8)				
Patient age in examination (h, IQR)	3.0 (2.0–15.0)	28.5 (21.8–36)	90.0 (47.0–140)	
PDA open (*n*)	3	1	0	
PDA size (mm, IQR)	0 (0–1.8)	0 (0–0)	0 (0-0)	
PDA direction [L-R, (*n*)]	3	1	0	
Left Ventricle SF	35.4 ± 1.2	34.0 ± 1.8	34.3 ± 1.8	0.22
PDA in Bi-direction (*n* = 28)				
PDA open (*n*)	28	25	16	
Patient age (h, IQR)	2.0 (1.1–2.0)	22 (22.0–23.0)	46.5 (46.0–52.6)	
PDA size (mm, IQR)	4.0 (3.3–4.9)	2.2 (1.1–3.6)	1.5 (0–2.6)	
PDA direction [L-R/Bi-direction/R-L(*n*)]	0/28/0	14/14/0	22/4/2	
Left Ventricle SF	36.0 ± 0.8	37.9 ± 0.9	35.4 ± 0.7	0.24
PDA in R-L group (*n* = 23)				
PDA open (*n*)	23	23	19^a^	
Patient age (h, IQR)	2.0 (1.5–3.0)	22.3 (21.6–23.0)	47.0 (46.0–60.0)	
PDA size (mm, IQR)	5.0 (4.0–5.2)	4.0 (2.4–5.0)	3.3 (0–4.1)	
PDA direction [L-R/Bi-direction/R-L(*n*)]	0/0/23	3/8/12	4/10/5	
Left Ventricle SF	33.1 ± 1.3	34.1 ± 1.4	33.3 ± 1.5	0.24

PDA, Patent ductus arteriosus.

^a^ There were two deaths before surgery and two PDA closures.

### 3.4. Comparison of patient outcomes based on PDA shunting direction

The duration of ventilation time, length of hospital stays, and frequency of intubation were significantly different and increased progressively worse (*p *< 0.05, Table 3) in patients with left-to-right (L-R) shunting, bi-directional shunting, and right-to-left (R-L) shunting (excluding nine non-survivors), respectively. The age at surgery was not different among these groups. Most patients who survived showed PDA closure within two weeks; however, two cases exhibited persistent PDA and underwent surgical PDA ligation. None of the discharged patients showed evidence of pulmonary hypertension, and supplementary oxygen was not required in any of these cases.

**Table 3:** Comparison of patient outcomes based on the directionality of PDA shunting.

	No PDA + L-R shunting (*n* = 8)	Bi-directional shunting (*n* = 28)	R-L shunting (*n* = 14)	*p*-value
Surgery age (days, median, IQR)	3.0 (1.0–5.0)	1.0 (1.0- 2.0)	1.0 (1.0–2.0)	1.000
Ventilation time (d)	2.9 ± 2.8	9.1 ± 6.6	17.2 ± 10.9	<0.001
Length of stay (d)	25.9 ± 7.3	30.5 ± 13.0	44.9 ± 14.4	0.001

PDA, Patent ductus arteriosus.

### 3.5. Comparison of Non-survivors and survivors in the R-L group

Newborns with R-L shunting determined within 24 h after birth were divided into survivors (*n* = 14) and non-survivors (*n* = 9). Of the 9 non-survivors, 7 patients continued to display R-L shunting before surgery, while the other 2 patients died before surgery following medical treatment. Among the 14 survivors, most patients (*n* = 13) showed a shift in the PDA direction to bi-directional or L-R shunting from R-L shunting, and the size of the PDA decreased compared to the measurement taken after birth, indicating a positive response to medical intervention.

The results revealed a significant difference between non-survivors and survivors in terms of the PDA shunt direction, PDA size, and left ventricle shortening fraction before surgery (Table 4). Specifically, non-survivors with persistent R-L shunting after medical treatment had larger PDAs and lower left ventricle shortening fraction before surgery compared to the survivors. In contrast, the survivors' PDAs changed to bi-directional or L-R shunting, and the PDA size became smaller than that observed after birth. These findings suggest that the persistence of R-L shunting after medicine treatment may be associated with a worse prognosis in newborns with congenital diaphragmatic hernia.

**Table 4:** Comparison between the non-survived and survived cases in R-L group.

	Non-survivor	Survivor	*P*-value
Diaphragmatic defect side (Left/Right, *n*)	4/5	10/4	0.383
PDA size after birth (mm)	5.1 ± 0.8	4.5 ± 1.2	0.153
PDA size before surgery (mm)	5.7 ± 1.4	3.6 ± 1.1	0.001
PDA direction before surgery (R-L/other direction)	8/1	2/12	0.003
Shortening fraction before surgery (%)	29.6 ± 6.3	34.4 ± 4.5	0.04

PDA, Patent ductus arteriosus.

### 3.6. Correlation between outcomes and PDA direction and size

Statistical analysis revealed significant correlations between patient outcomes and pre-operative PDA shunt direction (*R* = 0.571, *p* < 0.001), as well as PDA direction after birth (*R* = 0.463, *p* < 0.001). Additionally, the PDA size after birth and before surgery, gestational age of diagnosis, and left ventricle shortening fraction before surgery were also significantly correlated with patient outcomes (*R* = 0.325, 0.337, 0.329, 0.307, respectively, *p* < 0.05) (Table 5).

**Table 5:** Correlation of patient outcomes with the change of PDA size and direction before surgery.

	Correlation	*P*-value
PDA shunt direction after birth (24 h)	0.463	<0.001
PDA shunt direction before surgery	0.571	<0.001
PDA size after birth (24 h, mm)	0.325	0.012
PDA size before surgery (mm)	0.337	0.009
PDA size after surgery (mm)	0.053	0.692
Gestational age of diagnosis (w)	0.329	0.011
Diaphragmatic defect position (*n*)	0.158	0.233
Shortening fraction before surgery (%)	0.307	0.018

PDA, Patent ductus arteriosus.

Binary logistic regression analysis identified the direction of the preoperative PDA shunt as the most relevant factor among these relationships (*p* = 0.009, odds ratio 20.6, confidence interval 2.2–196.1).

## 4. Discussion

This study provided important insights into the factors influencing outcomes for CDH patients, particularly the role of PDA in the development and progression of PH. The findings confirm the significant impact of PH on morbidity and mortality in CDH patients, as well as the importance of early assessment of pulmonary vascular resistance. The study also identified several key factors that contribute to outcomes for CDH patients, including PDA shunting direction and size before surgery, gestational age at diagnosis, and shortening fraction before surgery. The direction of PDA shunting pre-operatively was found to be an independent risk factor for adverse outcomes, highlighting the need for careful monitoring of PH status on postnatal echocardiography.

Echocardiography is commonly used to assess PH, especially in CDH patients and it not only assessed tricuspid regurgitation, patent ductus arteriosus (PDA), but also measured LV eccentricity index (12), pulmonary artery acceleration time (13), cardiac function (16–18). However, when there is cardiovascular communication, the directionality of shunting can also reveal information about the relative resistance of the pulmonary and systemic vascular beds or the relative compliance of the right and left ventricles. The directionality of shunting across the PDA can provide valuable information about the relative resistance of pulmonary and systemic vascular beds or the relative compliance of the right and left ventricles, allowing for more accurate measurement of systolic pulmonary artery pressure (SPAP). The directionality of flow across the PDA could be regarded as an indicator of the pressure gradient between the aorta and pulmonary artery. Left-to-right shunting indicates sub-systemic SPAP and allows for quantitative measurement of SPAP. In contrast, right-to-left shunting indicates supra-systemic SPAP, while bidirectional shunting indicates near-systemic SPAP (19). Assessment of SPAP based on PDA direction and flow is more accurate than the peak TR jet velocity method (7, 10), which is often inaccurate in children (20). In addition, only 38% of appropriate TRs were detected (10), compared to a rate of 52.5% in our study. While the PDA was examined in 91.5% of subjects on the first day after birth in our group. Also, Kipfmueller et al. found a PDA was visible in 97%, 72%, and 32% and a tricuspid regurgitation in 62%, 61%, and 30% on day of 2, 5–7, respectively (13). And PDA flow patten changed after the medical treatment before the surgery. Therefore, the assessment of the PDA is feasible at the first 48hs after birth and more accurate for assessing PH for CDH patients, particularly in situations where tricuspid regurgitation is not detectable.

General practice recommendations in CDH include obtaining an echocardiography within 24–48 h of birth to assess pulmonary vascular resistance and left ventricle function (15, 21). Additionally, previous studies have defined the first 48 h of delivery as a critical time frame for initial CDH-PH evaluation (16, 22). A recent analysis of a registry comprising over 1,400 CDH cases revealed that moderate or severe pulmonary hypertension (PAp > 2/3 systemic pressure) within the first 48 h of life was associated with an elevated risk of death or oxygen therapy at 30 days (23). Approximately 85% of CDH newborns manifest pulmonary hypertension within the first 48 h of life, with limited treatment options currently available (23, 24). Additionally, Leyens et al. reported the dynamics of PH in the first 48 h of life at three defined times point of echocardiography and observed a fluctuation in PH severity in approximately two third of all patients (25). We had similar results with those reports, while we not only examined every patient and had a primary diagnosis for the first several hours after birth, but measured changes of PH after medical treatment on the second day or before the surgery within 24 h. On the second echo, the PDA directionality of shunting and diameter changed, which predicted outcomes in this patient population from our results. Given our findings, patients with a closed PDA or a L-R shunting at the first 24 h echocardiography had a shorter hospital length of stay and ventilation time than patients with a patent PDA with bi-directional or R-L shunting, and PDA shunting converting to bi-directional or L-R shunting or a smaller diameter on the second echocardiography before the surgery illustrated a better outcome comparison between non-survivors and survivors in the R-L group. There was another similar report about that a VTI ratio between R-L and L-R shunting of PDA <1.0 was a valuable threshold to identify high-risk in CDH neonates (26). While non-survived patients with R-L PDA shunting all exhibited larger PDA sizes and continuous R-L shunting even after medical treatment. Furthermore, the results of correlation and logistic regression for the first time suggested that the PDA shunt direction and size may serve as useful predictors of patient outcomes in cases of R-L shunting. Specifically, infants with larger PDAs and persistent R-L shunting may be at higher risk for adverse outcomes, whereas those with smaller PDAs and a change in shunt direction may have better prognoses. Monitoring these changes could provide additional information on the rapid changes in pulmonary arterial pressure. So in CDH infants, the PAp level within the first 48 h of life has been shown to predict patient outcomes. Our study adds to this body of evidence by demonstrating that changes in PDA shunt directionality and diameter may also serve as useful prognostic indicators.

## 5. Limitation

One of the limitations of this study is its relatively small sample size, which may restrict the generalizability of the results. Future studies with larger sample sizes may be needed to validate our findings and determine their applicability to other CDH populations. Another limitation of this study is its retrospective nature, which may have introduced selection bias and confounding variables that were not accounted for in the analysis. Additionally, Arterial blood pressure values were not always recorded during every echocardiography examination, so the results were not calculated. Again, patients with L-R shunting were significantly older at the third examination compared to those with bidirectional or R-L shunting and this might have influenced the observed shunting pattern. Furthermore, this study was conducted at a single center, and the generalizability of the findings to other centers and patient populations may be limited. Finally, the use of different treatment strategies and variations in clinical practices among different centers may have impacted the study results.

## 6. Conclusion

In conclusion, our study highlights the significance of assessing the direction and diameter of PDA shunting in newborns with CDH within 48 h of birth, especially the changes of PDA before surgery. We found that R-L PDA shunting was significantly associated with higher mortality, longer ventilation time, and hospital length of stay, while a closed PDA, L-R, or bi-directional shunting was not associated with PH at discharge and had better outcomes. Our findings also suggest that PDA shunt direction and diameter changes before surgery can predict inpatient outcomes, with unchanged PDA direction or increased PDA diameter associated with worse outcomes.

Early echocardiogram evaluation can serve as a prognostic factor for CDH management and help clinicians identify high-risk patients who may require more aggressive monitoring and treatment. Further studies with larger sample sizes and long-term follow-up are needed to confirm the utility of PDA shunting direction and diameter as a prognostic factor in CDH management.

## Data Availability

The raw data supporting the conclusions of this article will be made available by the authors, without undue reservation.
